# EXPRESSION OF CONCERN: Photodynamic Therapy with 5‐Aminolaevulinic Acid and DNA Damage: Unravelling Roles of p53 and ABCG2

**DOI:** 10.1111/cpr.70242

**Published:** 2026-06-04

**EXPRESSION OF CONCERN**: I. Postiglione , F. Barra , S. M. Aloj , and G. Palumbo , “Photodynamic Therapy with 5‐Aminolaevulinic Acid and DNA Damage: Unravelling Roles of p53 and ABCG2,” Cell Proliferation 49, no. 4 (2016): 523–538, 10.1111/cpr.12274.27389299 PMC6496272

This Expression of Concern is for the above article, published online on 07 July 2016 in Wiley Online Library (wileyonlinelibrary.com), and has been issued by agreement between the journal Editor‐in‐Chief, Qi Zhou; and John Wiley & Sons Ltd. The Expression of Concern has been agreed due to concerns raised by third parties. Specifically, a cell in Figure 4E within the 20 Jcm^−2^ panel was found to have been duplicated and shown at a slightly different magnification. In their clarification, the authors explained that the Comet Score analysis panels are composite figures created by selecting four representative cells per experimental condition, which may originate from different and non‐contiguous microscopic fields. They further acknowledged that a human error had resulted in the same representative cell being included twice within one panel.

Because the panels do not demarcate or indicate separate fields, this method of presentation was deemed inappropriate. In addition, due to the time elapsed since publication, the authors were unable to retrieve the original data to correct the error by providing an alternative and appropriate image. However, as the issues were found not to compromise the article's conclusions, the journal has decided to issue an Expression of Concern to inform and alert the readers.

